# Renal Dysfunction among HIV-Infected Patients on Antiretroviral Therapy in Dar es Salaam, Tanzania: A Cross-Sectional Study

**DOI:** 10.1155/2020/8378947

**Published:** 2020-10-08

**Authors:** Oswin Mwemezi, Paschal Ruggajo, Jonathan Mngumi, Francis F Furia

**Affiliations:** ^1^School of Medicine, Muhimbili University of Health and Allied Sciences, Dar es Salaam, Tanzania; ^2^Renal Unit, Muhimbili National Hospital, Dar es Salaam, Tanzania

## Abstract

**Background:**

HIV-associated renal dysfunction is common among infected patients; the growing burden of this condition may be partly accounted for by improved survival attributed to sustained viral suppression with antiretroviral therapies (ART). Some ART regimens are nephrotoxic and may potentially contribute to renal dysfunction observed in these patients. This study aimed at investigating the prevalence of renal dysfunction among people living with HIV (PLHIV) on ART attending the care and treatment clinic (CTC).

**Methods:**

A cross-sectional study was conducted between June and October 2019 among adults living with HIV on ART for 6 months or more attending CTC at Muhimbili National Hospital in Dar es Salaam, Tanzania. A total of 287 participants were screened for proteinuria and microalbuminuria using the Cybow 300 urine analyzer. Serum creatinine was tested for all participants, and it was used to estimate glomerular filtration rate (eGFR) using the CKD-EPI formula.

**Results:**

Out of 287 participants (72.1% female, mean age ± SD: 46.7 ± 10.6 years), about one-third (32.8%) had eGFR less than 90 ml/min, whereas 7% had eGFR less than 60 ml/min. Microalbuminuria and proteinuria were detected in 38.6% and 25.1% of participants, respectively. In the multivariate analysis, predictive determinants for renal dysfunction were higher viral loads (OR 2.5 (1.1–5.8), *p*=0.031), diabetes mellitus (OR 5.5 (1.6–18.6), *p*=0.006), and age above 60 years (OR 2.8 (1.0–7.3), *p*=0.041); however, this was not the case for serum CD4 counts (OR 1.25 (0.7–2.3), *p*=0.46).

**Conclusion:**

High prevalence of renal dysfunction among PLHIV on ART was noted in this study. Viral loads above 1000 cp/ml and diabetes mellitus were noted to be associated with increased risk for renal dysfunction.

## 1. Background

People living with HIV globally were estimated to be about 37.9 million by 2018, of which 20.6 million were in Eastern and Southern Africa [1]. In Tanzania, around 1.4 million people were infected with HIV by 2019 [2].

PLHIV are at risk of developing renal dysfunction; this may be due to HIV infection, especially poor viral suppression, antiretroviral toxicity, and other risk factors including old age, female sex, diabetes, hypertension, injection drug use, smoking, and history of previous renal insults such as acute kidney injury [3–6]. The burden of renal dysfunction among HIV patients differs across the globe; various studies conducted in sub-Saharan Africa using different methods to define kidney disorders have reported a wide spectrum of prevalence rates ranging from 6% to 76% [5–7].

High prevalence of 76% of renal dysfunction was reported among ART-naive HIV patients in Tanzania [5]. With stable antiretroviral therapy (ART) programs, patients are expected to live longer and may be exposed to age-related noncommunicable disorders including renal disorders. There are limited data on the magnitude of renal dysfunction among patients on ART in Tanzania; however, studies from Nigeria and South Africa have documented increased prevalence of CKD among HIV patients on ART [3, 8]. This study was therefore carried out to give a snapshot of the burden of renal dysfunction and its predictors among HIV patients on ART (for at least six months) in Tanzania.

## 2. Methods

### 2.1. Study Design and Setting

This was a hospital-based cross-sectional study conducted between June and October 2019 at Muhimbili National Hospital (MNH) based in Dar es Salaam. MNH has a bed capacity of more than 1500 and is the largest referral hospital in Tanzania; it serves as a teaching hospital for Muhimbili University of Health and Allied Sciences (MUHAS). It provides HIV services for both inpatients and outpatients and serves a total of 1400 patients per month in the clinics.

### 2.2. Study Population and Sample Size

HIV-infected adults aged 18 years or who were attending the clinic at MNH and had been using ART for 6 months or longer were eligible for this study. Sample size for this study was calculated based on the prevalence of renal dysfunction reported among ART-naive HIV-infected patients by Msango et al. in Mwanza, Tanzania [5]. Leslie and Kish formula was used giving a minimum sample size of 280 participants. A total of 287 participants were subsequently recruited for this study.

Participants were identified using a simple random sampling technique, whereby the identification numbers of patients that attended the clinic in a specific day were obtained from the appointment book, written on pieces of papers, and a rotary carried out to randomly obtain ten participants each day. The procedure was repeated on each clinic day until the sample size was reached.

### 2.3. Data Collection

A structured questionnaire was used for data collection, which included sociodemographic and clinical information and physical examination findings. History of ART use and regimen, recent viral load, and CD4 count were recorded in the questionnaire from the care and treatment clinic record card. Blood pressure was measured using the standard, semiautomated, and calibrated Omron blood pressure machine. This was taken on the left arm after the patient had taken rest for about five minutes; the average of two readings was taken as the blood pressure of the patient. Body weight was measured using a standard weighing scale while patient had not worn shoes, and a stadiometer was used to measure the height of participants to the nearest centimeter by a well-trained CTC nurse; BMI was then calculated from the readings by the principle investigator.

Each participant was requested to provide the urine specimen, and 5 ml of blood was drawn aseptically from their left antecubital fossa by a trained phlebotomist. Urine specimen was stored temporarily at 4°C and thereafter transported within 30 minutes from the collection site to the laboratory using a biohazard cool box for testing. A drop of blood was used for checking RBG, and the blood specimen was sent to the MNH pathology laboratory.

### 2.4. Laboratory Procedures

Blood glucose was checked using a recent calibrated GlucoPlus machine, and the history of diabetes mellitus and use of antidiabetics were ascertained.

Serum creatinine was measured by using COBAS INTEGRA® 400 plus by Cobas-Roche for all the study participants, and each participant's eGFR was calculated using CKD-EPI.

Urine was analyzed for protein, albumin, albumin-to-creatinine ratio, nitrite, red blood cells, white blood cells, and pH using the urine chemistry analyzer (Cybow reader 300) and 12C Cybow strips.

### 2.5. Data Analysis

All questionnaires were checked for completeness, and data were entered into Statistical Package for Social Sciences version 20. Data cleaning was carried out by consistence checks. Analysis was carried out using the same software; summary statistics were determined as means with standard deviation, median, and range for continuous data and frequencies with percentages for categorical data. Continuous data were analyzed using Student's *t*-test, while chi-square and Fisher's exact tests were used for categorical data. Association between independent and dependent variables was determined using univariate analysis, and all variables that are traditionally known to be risk factors for renal dysfunction were further analyzed in multivariate analysis using binary logistic regression models to adjust for confounders. A two-tailed *p* value of <0.05 was considered statistically significant, and the Hosmer–Lemeshow test was used to determine the goodness of fit for the final logistic regression model.

### 2.6. Study Variables

The main outcome variable was the presence of renal dysfunction which was defined by eGFR below 60 ml/min/1.73 m^2^ and/or presence of both proteinuria and microalbuminuria.

Albuminuria was defined by the presence of albumin-to-creatinine ratio (ACR) ≥30 mg/g, and proteinuria was defined by ≥ + 1 proteinuria.

The independent variables were viral load and CD4 levels, hypertension, diabetes mellitus, age, marital status, and sex.

Hypertension in this study was defined based on the previous diagnosis of hypertension or blood pressure of 140/90 mmHg or more on two readings taken at least 5 minutes apart [9].

Diabetes mellitus (DM) in this study was defined based on the previous diagnosis of DM or the presence of fasting plasma glucose (FPG) level equal to or greater than 7.0 mmol/L or random plasma glucose level equal to or greater than 11.1 mmol/L in a patient with classic symptoms of hyperglycemia [10].

## 3. Results

A total of 1,040 patients attended the clinic during the study period; 311 participants were randomly selected for the study. Among those sampled, 24 patients were excluded (5 had urinary tract infection, 8 had missing serum creatinine laboratory results, and 11 did not consent), and 287 participants were included in the final analysis.

### 3.1. Demographic and Clinical Characteristics

Two hundred and eighty-seven participants were recruited into this study out of which 207 (72.1%) were female; mean age of participants was 46.7 ± 10.6 with an age range of 20–73 years. Two hundred and forty-nine participants (86.8%) had lower education levels, and 129 (45.0%) were living with a partner.

A majority of participants 248 (87%) were using the tenofovir-based ART regimen. Seventy percent had CD4 levels above 350 cells/ml, and almost ninety percent of participants (257/287) had good viral suppression (VL ≤ 1000 copies/ml).

Hypertension was present in 24% of the study participants, and not all had been diagnosed, and 4% had diabetes mellitus previously diagnosed but with poor blood sugar control (Table 1).

### 3.2. Prevalence of Renal Dysfunction

Renal dysfunction was detected in a quarter (24.7%) of the study participants. About twenty-five percent of all the participants (72/287) had proteinuria, and microalbuminuria was present in 38.6% (11/287) of the participants. Sixty-one participants (21.3%) had both proteinuria and microalbuminuria. Around seven percent (20/287) had eGFR below 60 ml/min/1.73 m^2^.

### 3.3. Predictors of Renal Dysfunction

Factors that were noted to influence the occurrence of renal dysfunction included higher viral load levels, diabetes mellitus, and old age (above 60 years). Participants with diabetes mellitus had five-fold increased likelihood of getting renal dysfunction compared to those without diabetes (OR 5.5, 95% CI [1.6–18.6], *p*=0.006). Similarly, participants with viral load levels 1000 cp/ml and above had twice increased odds for renal dysfunction than those who had viral load below 1000 cp/ml (OR 2.5, 95% CI [1.1–5.8], *p*=0.03).

Participants with age above 60 years were more likely to get renal dysfunction than those who had age below 60 years.

CD4 count and type of ART used did not show any association of occurrence of renal dysfunction in this study (Table 2).

## 4. Discussion

Renal dysfunction was noted to be common among HIV-infected patients on antiretroviral therapy. About a quarter (71/287) had renal dysfunction; higher viral loads, diabetes mellitus, and older age above 60 years were associated with increased risk of renal dysfunction.

The high prevalence of renal dysfunction (24.7%) from this study could be attributed to the strict criteria used in this study, and lower threshold in screening for renal dysfunction enables early detection of the patients and hence room for providing earlier interventions. Higher magnitudes (53–76%) of renal dysfunction were reported among ART-naive patients compared to our findings [5, 11]. Most of our participants had good viral suppression, indicating possibly ART medications may be protective of renal dysfunctions.

Microalbuminuria is an early sign of progressive cardiovascular and renal disease in patients with various risk factors, and proteinuria is linked with faster progression of HIV as well as kidney diseases [11]. Higher prevalence rates of microalbuminuria and proteinuria were noted in this study compared to other similar studies [7, 12], showing the need for routine screening of HIV-infected patients including those with good viral suppression. Timely treatment of microalbuminuria and proteinuria is reported to retard renal progression and reduce endothelial and cardiovascular risks [11].

Diabetes mellitus (DM) was noted in 4.2% of the study participants, and half of these participants had renal dysfunction. Similar findings were reported by the study done in rural South Africa among HIV patients on ART [13]. Use of ART for long duration is reported to cause increased insulin resistance, and diabetes mellitus causes the damage of the glomeruli leading to renal dysfunction [14]. Almost all patients with DM in our study were previously diagnosed but had poor blood sugar control; this might have also contributed to their increased risk for renal dysfunction. Regular monitoring and control of blood sugar among HIV patients with DM could therefore reduce their risk for renal impairment.

High viremia is established for HIV-associated nephropathy (HIVAN) [7]. Although most of the participants in our study had good viral suppression, however, it was observed that those with viral load levels higher than 1000 cp/ml were associated with increased odds of renal dysfunction than their counterparts with viral load levels below 1000 cp/ml. This is in keeping with results from other studies [15], showing the need to make sure that all HIV patients on ART have good viral suppression so as to protect them from HIV-associated nephropathy.

This study is one among few studies done in Tanzania, looking at renal dysfunction among HIV patients on ART. It has given a snapshot on the burden of renal dysfunction and associated risk factors among HIV patients stable on ART. This study also followed the standard definitions of renal impairment, hypertension, and diabetes mellitus.

It was a cross-sectional study carried out in one center which may not give generalizable prevalence of renal dysfunction in HIV patients in Tanzania but rather those attending this clinic; in this setting, a multicenter study across the four zonal hospitals would be very helpful.

## 5. Conclusion

High prevalence of renal dysfunction among Tanzanian PLHIV and stable on ART as demonstrated in our study with higher viral loads and diabetes mellitus being the risk factors prioritizes continuous routine viral load monitoring among all PLHV and proper control of those with DM.

We recommend incorporating screening for renal dysfunction and diabetes among HIV patients in the HIV care and treatment clinic programs. This will enable early detection, treatment, and monitoring of HIV patients for such complications.

## Figures and Tables

**Table 1:** Demographic and clinical characteristics of HIV patients on antiretroviral therapy at Muhimbili National Hospital (*N* = 287).

Characteristics	*n* (mean ± SD) Median, range	%
*Gender of the respondents*		
Female	207	72.1
*Education level*		
No formal education	5	1.7
Lower education level	249	86.8
Higher education level	33	11.5
*Marital status*		
Single	71	24.7
Married/cohabiting	129	45.0
Divorced	87	30.3
*ART regimen*		
Tenofovir-based ART regimens	249	86.8
Non-tenofovir-based ART regimens	38	13.2
*Age in years* (mean ± SD)	46.51 ± 10.8	
*CD*4 *count* (mean ± SD)	516 **±** 303	
Median	464	
Range	6–1839	
*Viral load* (mean ± SD)	170284 **±** 2328694	
Median	50	
Range	20-39237898	
*Hypertensive*	71	24.7
Systolic blood pressure (mean ± SD)	122 ± 21	
Diastolic blood pressure (mean ± SD)	78 ± 13	
*Diabetic*	12	4.18
Random blood glucose (mean ± SD)	5.8 ± 2.5	
Median	5.3	
Range	2–23	
*Albumin-to-creatinine ratio (ACR)* (mean ± SD)	42 ± 52.5	
Median	20	
Range	2–300	
*eGFR value* (mean ± SD)	97.5 ± 24	
Median	101	
Range	6–154	

**Table 2:** Factors predictive of renal dysfunction among HIV patients on antiretroviral therapy at Muhimbili National Hospital (*N* = 287).

Characteristics	Renal dysfunction, *n* = 71 (24.7%)	No renal dysfunction, *n* = 216 (75.3%)	Total (*N*)	Univariate OR, 95% CI, *p* value	Multivariate OR, 95% CI, *p* value
*Age*					
Less than or equal to 60 years	62 (23.4)	203 (76.6)	263	Reference	
More than 60 years	9 (40.9)	13 (59.1)	24	**2.2, (0.9–5.5), 0.07**	**2.7,(1.04–7.3), 0.04**
*Gender*					
Male	23 (28.8)	57 (71.2)	80	Reference	
Female	48 (23.2)	159 (76.8)	207	0.7, (0.4–1.3), 0.32	0.95, (0.5–1.8) 0.9
*ART regimen*					
Non-tenofovir-based	13 (34.2)	25 (65.8)	38	Reference	
Tenofovir-based	58 (23.3)	191 (76.7)	249	0.6, (0.3–1.2), 0.15	0.6, (0.3–1.35) 0.23
*CD*4 *count*					
Less than 350 cells/mm^3^	27 (31.4)	59 (68.6)	86	1.6, (0.9–2.8), 0.09	1.25, (0.67–2.3) 0.5
350 cells/mm^3^ and above	44 (21.9)	157 (78.1)	201	Reference	
*Viral load*					
Less than 1000 cp/ml	59 (23)	198 (77)	257	Reference	
1000 cp/ml or more	12 (40)	18 (60)	30	**2.2, (1.02–4.9), 0.045**	**2.5, (1.09–5.8), 0.03**
*Blood pressure*					
Normal BP	52 (24.0)	165 (76)	2017	Reference	
Hypertensive	19 (27.1)	51 (72.9)	71	1.2, (0.64–2.1), 0.6	0.94, (0.5–1.8), 0.86
*Blood sugar*					
Nondiabetic	64 (23.3)	211 (76.7)	275	Reference	
Diabetic	67 (58.3)	5 (41.7)	12	**4.6, (1.4–15), 0.01**	**5.5, (1.6–18.6), 0.006**

## Data Availability

The datasets generated and analyzed during this study are not publicly available to maintain participant confidentiality. The data are however available from the corresponding author upon reasonable request.
